# Supplementary material for: MetaRibo-Seq measures translation in microbiomes
Source: Nat Commun. 2020 Jun 29;11:3268. doi: 10.1038/s41467-020-17081-z (PMC7324362; doi:10.1038/s41467-020-17081-z)
Supplement: Supplementary file 10 — Supplementary Data 7 [file 41467_2020_17081_MOESM10_ESM.zip › File2/Confidence_High_Taxonomy/178579_out.krona.html]

Javascript must be enabled to view this page.

members
magnitude
magnitudeUnassigned
count
unassigned
taxon
rank

178579\_out

6

superkingdom
6
2

6
phylum
1239

6
class
186801

6

SRS011302\_contig\_number\_29527SRS1041031\_contig\_number\_10714SRS1041033\_contig\_number\_3515SRS104636\_contig\_number\_31889SRS147139\_contig\_number\_43353SRS892882\_contig\_number\_contig-100\_12242.54480
species
2044939
